# A new and sensitive method for quantitative determination of helium in human blood by gas chromatography–mass spectrometry using naturally existing neon-21 as internal standard

**DOI:** 10.1007/s11419-018-0437-6

**Published:** 2018-08-13

**Authors:** Akira Tsujita, Hidehiko Okazaki, Asami Nagasaka, Akinaga Gohda, Mitsushi Matsumoto, Toshiro Matsui

**Affiliations:** 1 Forensic Science Laboratory, Fukuoka Prefectural Police Headquarters, 7-7 Higashikoen, Fukuoka, 812-8576 Japan; 2 Department of Bioscience and Biotechnology, Faculty of Agriculture, Graduate School of Kyushu University, 744 Motooka, Fukuoka, 819-0395 Japan

**Keywords:** Helium analysis, Neon-21 as internal standard, Whole blood, GC–MS in SIM mode, Asphyxia, Inhalation suicide

## Abstract

**Purpose:**

In this study, we proposed a new sensitive quantitative method for detecting helium in human blood by gas chromatography–selected-ion monitoring (SIM)-mass spectrometry (GC–SIM-MS) using naturally existing neon-21 in air as internal standard (IS).

**Methods:**

GC–SIM-MS analysis was performed on a double TC-Molsieve 5A capillary column (total length 60 m) for the separation of permanent gases by a single-run experiment. By using hydrogen as the carrier gas, the analyte (helium) and IS (neon-21) were separated on the double column, and detected at *m/z* 4 and 21, respectively. The ratio of the peak area of helium-to-neon-21 was used for obtaining the calibration curve for helium determination.

**Results:**

The limits of detection and quantification of helium under the present GC–SIM-MS conditions were as low as 1.8 and 6.0 ppm, respectively. The proposed GC–SIM-MS method also showed high repeatability with relative standard deviation at 1.3–5.1%, indicating that the use of neon-21 as IS was valid for reliable helium assays. The successful quantification of helium in the headspace of vacuum blood collection tubes containing the whole blood from four humans who died of helium inhalation was achieved using the proposed neon-21-aided GC–SIM-MS method; the values obtained for helium were 24–497 ppm.

**Conclusions:**

The proposed GC–SIM-MS method in combination with the naturally existing neon-21 as IS is most recommendable for quantitative assays of helium in biological samples because of its simplicity and extremely high sensitivity.

## Introduction

Helium is considered as an inert gas that has no flammability. It has been reported that deaths due to suffocation by excess helium inhalation are becoming an increasing serious social issue [1–7]. However, the lack of an appropriate analytical assay for the helium in the biological samples results in the difficulty for clarifying the unequivocal cause of death in the helium-related cases.

Gas chromatography–thermal conductivity detection (GC–TCD) has been commonly used as a convenient method for helium assays so far. Some studies have reported the detection of helium accumulated in the organs (lung, stomach, brain, liver, and trachea) and blood by GC–TCD [7–11]. However, the GC–TCD method suffers from poor sensitivity and selectivity for helium detection [12]. Although headspace analysis of the helium in the blood during autopsy may solve the problem of insufficient detection [8, 9], it still suffers from poor reproducibility for the helium assay due to the unstable volatility of the helium found in the blood matrix. Norimine et al. [10] improved the poor GC–TCD detection of helium by increasing the helium volatility in the headspace using a reduced-pressured vial. To date, gas chromatography–mass spectrometry (GC–MS) has been extensively used for the selective detection of helium in biological samples including the lungs, stomach, trachea, and blood. Auwaerter et al. [13] and Musshoff et al. [14] qualitatively assayed helium using nitrogen and hydrogen as carrier gases, respectively, on a nonpolar capillary column by the selected-ion monitoring (SIM) GC–MS technique. Alternatively, Malbranque et al. [15] proposed a quantitative GC–SIM-MS assay of the helium in the organs with the aid of an external standard (nitrous oxide; N_2_O). Although the use of N_2_O as a standard in GC–SIM-MS analysis might be useful for quantitative helium assay, it required a mixing of the headspace gas with N_2_O in another vial owing to its high solubility in biological samples, causing difficulty in direct helium assay in the target headspace samples.

Because of the aforementioned disadvantages of the reported GC–SIM-MS methods for complex biological samples, in this study, we tried to develop an internal standard (IS)-aided GC–SIM-MS method for quantitative helium assays. Blood was targeted for the present assay, since the blood of the deceased is the first priority matrix for judging the asphyxiation by helium inhalation. The application of the neon-21 naturally existing in air (0.049 ppm [16, 17]) as the IS, but not as the external standard, to the present GC–SIM-MS assay is described in this article.

## Materials and methods

### Materials

Helium (99.995% purity), argon (99.995%), and nitrogen (99.995%) were purchased from Taiyo Nissan Co. (Osaka, Japan). Standard helium gas at a concentration of 100 ppm in nitrogen was obtained from GL Sciences (Tokyo, Japan). Helium with concentrations of 10–1000 ppm were prepared by diluting pure helium with air in an aluminum bag (1 L, GL Sciences) using a gas-tight syringe (SGE Analytical Science, Melbourne, Australia). The sample gas (1.0 mL) was injected into a GC–SIM-MS system with a gas-tight syringe fitted with a push-pull valve (1 mL-volume of syringe, SGE Analytical Science).

The commercially available human whole blood was purchased from Cosmo Bio Co. (No. 12081545; Tokyo, Japan).

### GC–SIM-MS analysis

**Fig. 1:**
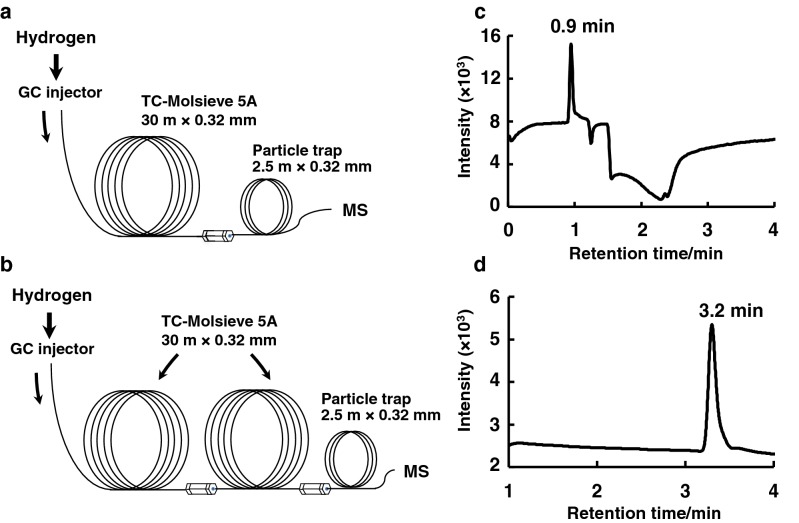
Chromatographic design of the **a** single column and **b** double column used for helium analysis. Selected-ion monitoring (SIM) chromatograms for the **c** single column and **d** double column using a helium standard sample (100 ppm) prepared in nitrogen. Conditions: capillary column, TC-Molsieve 5A (single column, 30 m × 0.32 mm; double column, 60 m × 0.32 mm); injection volume, 1.0 mL; oven temperature, 34 °C; monitoring mode, SIM (*m/z* 4)

GC–SIM-MS analysis was carried out on a Shimadzu GC–MS QP2010plus (Shimadzu, Kyoto, Japan). A TC-Molsieve 5A capillary column (30 m × 0.32 mm i.d., film thickness 30 μm, GL Sciences) was used for the separation. In this study, either a single (Fig. 1a) or double column (two connected TC-Molsieve 5A capillary columns of total length 60 m; Fig. 1b) was used for the GC–SIM-MS analysis. In single-column assay, a particle trap (2.5 m × 0.32 mm, GL Sciences) that prevents the mass detector from contamination was connected to the analytical column on the detector side using a capillary mini-union (GL Sciences; Fig. 1a). In double-column assay, two TC-Molsieve 5A capillary columns and the particle trap were connected in series (Fig. 1b). The GC conditions were as follows, except for the carrier gas: column temperature, 34 °C; split ratio, 2:1; purge flow rate, 3.0 mL/min; and injection temperature, 35 °C. The MS conditions were as follows: electron ionization (EI) mode; detector gain, 1.0 kV; ionization voltage, 70 eV; emission current, 150 µA; interface temperature, 150 °C; ion source temperature, 200 °C; monitoring ions, *m/z* 4 and 21 in the SIM for helium and neon-21, respectively.

Hydrogen, argon, and nitrogen were used as the carrier gases in this study. Pure hydrogen (99.9999%) was supplied by a hydrogen generator NMH-100 (Air Tech, Yokohama, Japan), and the column flow rate was set at 36 cm/s.

### Quantitative neon-21-aided GC–SIM-MS analysis of the helium in whole blood specimens

For quantitative neon-21-aided GC–SIM-MS analysis of helium in whole blood, the whole blood (ca. 2.0–8.9 mL) taken from the heart of the deceased was transferred to a 10-mL vacuum blood collection tube containing sodium heparin (Terumo Co., Tokyo, Japan) and refrigerated at 4 °C until analysis. Prior to the analysis, the blood tube was stood for 30 min at 25 °C. After achieving the equilibrium of the headspace phase at 25 °C, 1.0 mL of the headspace was injected into the GC–SIM-MS system. For neon-21-aided GC–SIM-MS analysis as part of the calibration experiments, helium gas samples diluted with air were used; because air contains a constant concentration of neon-21 (0.049 ppm), the air dilution method indicates that the fixed amount of neon-21 can be automatically spiked into the headspace as IS. The commercially available blood sample was used as the blank specimen.

## Results and discussion

### Optimization of the GC–SIM-MS conditions for helium quantification

In order to separate and detect inert helium gas by GC–SIM-MS using a single column, a capillary GC column, TC-Molsieve 5A, which was suitable for inert gas retention [8], was used. A middle-bore column with a 0.32-mm i.d. was used for the present headspace GC analysis. The single-column-attached GC–SIM-MS system (Fig. 1a) allowed the detection of helium (100 ppm in air) at a retention time of 0.9 min at *m/z* 4 (Fig. 1c). However, an unstable baseline obtained in the GC–MS conditions may result in less reproducible detection of helium. It seems likely that the variable pressure observed in the capillary column and/or reduced vacuum pressure in the MS system owing to the low viscosity of the carrier gas hydrogen (at 65 cm/s) might be associated with the unstable baseline behavior. To overcome this problem, a double-column separation system using two TC-Molsieve 5A capillary columns (total 60 m × 0.32 mm) was applied for helium detection (Fig. 1b). As shown in Fig. 1d, stable baseline behavior, together with the longer retention of helium at 3.2 min, was achieved using the present GC–MS method, probably because of the increased back-pressure in the double-capillary columns. In addition, the double-column separation system improved the peak shape of helium for quantitative assays.

**Fig. 2:**
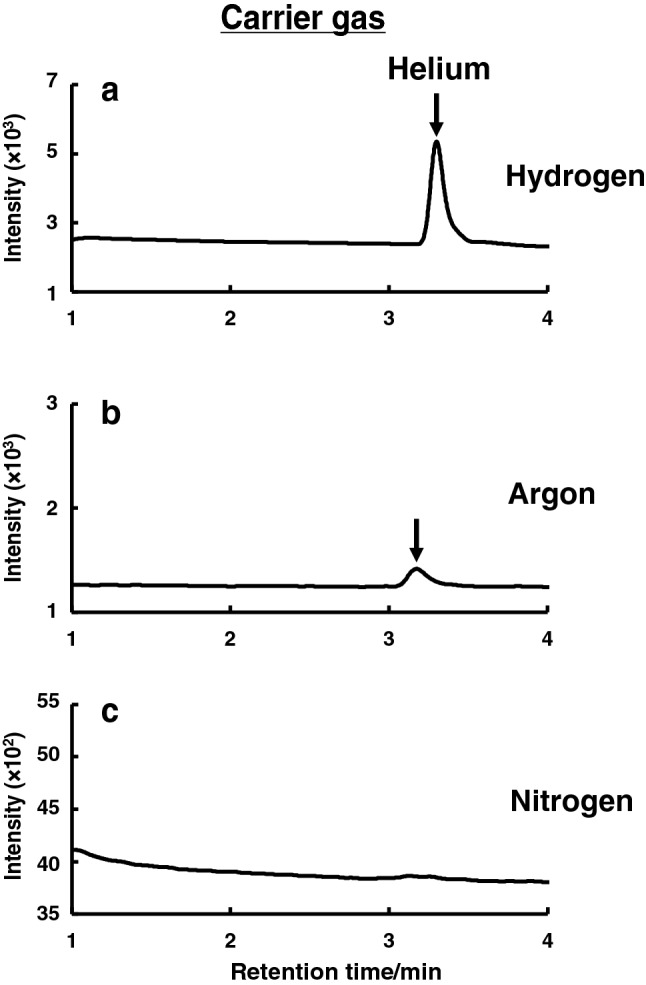
Effect of carrier gas on the helium (100 ppm) detection by gas chromatography–selected-ion monitoring-mass spectrometry (GC–SIM-MS). **a** Hydrogen, **b** argon, and **c** nitrogen. The double-capillary column TC-Molsieve 5A (60 m × 0.32 mm) was used. Other conditions were the same as specified in Fig. 1

Under the proposed double-column GC–SIM-MS conditions, the effects of carrier gases on the GC–SIM-MS detection of helium were investigated. Gases such as hydrogen, argon, or nitrogen were tested as the carrier gas in this study. Figure 2 shows the typical SIM chromatograms of helium (100 ppm) for each carrier gas at a flow rate of 36 cm/s. Among the three gases, hydrogen gave the highest peak intensity and a good peak shape as compared to the other two carrier gases. The poor MS detection of helium using argon or nitrogen as the carrier gas may be attributed to their high ionization cross sections, i.e., high molecular size and low ionization energy [18] that reduced the EI efficiency.

**Fig. 3:**
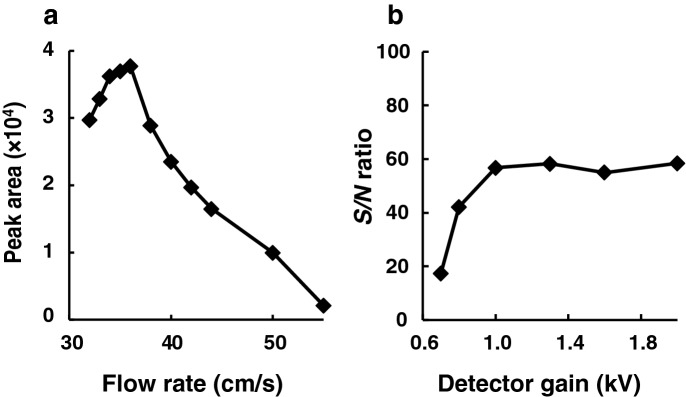
Effects of **a** the flow rate of the carrier gas hydrogen and **b** detector gain on helium detection by GC–SIM-MS

**Fig. 4:**
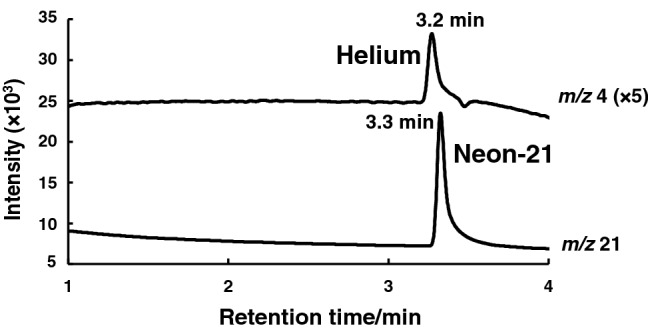
Typical SIM chromatograms for standard helium gas (100 ppm) and neon-21 in air. The double-capillary column was used, and SIM was carried out at *m/z* 4 and 21. Other conditions are the same as specified in Fig. 1

The effect of the flow rate of hydrogen (carrier gas) on the detection of helium (100 ppm) was then examined. As shown in Fig. 3a, the increase in the flow rate of hydrogen (from 32 to 65 cm/s) greatly affected the peak intensity of the helium detected; up to 36 cm/s, the peak intensity increased with increasing flow rate, while a gradual decrease in the peak area was observed with flow rates at  > 36 cm/s. Therefore, further experiments on helium detection were performed at 36 cm/s. Under the above conditions, the effect of MS detector gain on the detection was investigated. As shown in Fig. 3b, the detector gain greatly affected the sensitivity of helium detection; the signal-to-noise (S/N) ratio as an index of sensitivity increased with detector gain up to 1.0 kV, and thereafter plateaued. Thus, a 1.0-kV detector gain was used for the subsequent experiments. Under the optimized conditions, both helium (100 ppm in air) and neon-21 (0.049 ppm in air) were successfully detected by GC–MS-SIM at the retention times of 3.2 and 3.3 min, respectively (Fig. 4).

### Reliability of the method

**Fig. 5:**
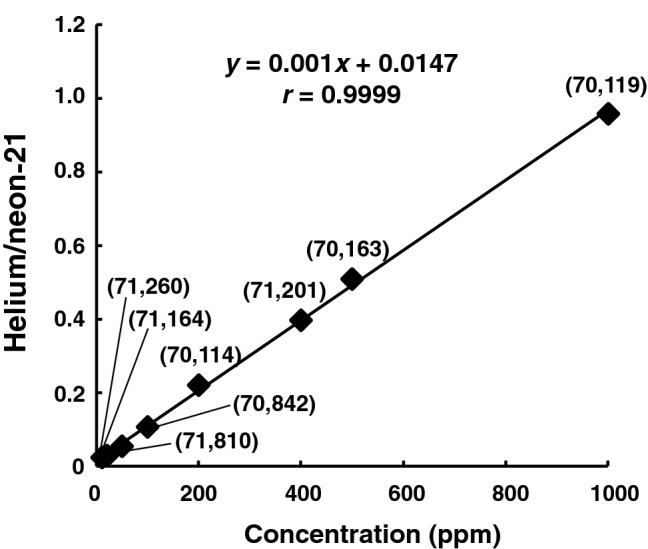
Calibration curve for detection of helium in air measured by GC–MS. The calibration range of helium was 10–1000 ppm. The peak area ratio of helium (*m/z* 4)-to-neon-21 (*m/z* 21) was used for the vertical axis. Each number inside the parentheses indicates the peak area units of neon-21

In the present GC–SIM-MS conditions, a concentration-dependent experiment on helium (10–1000 ppm in air) was then performed with the aid of neon-21 as IS to obtain the calibration curve. As shown in Fig. 5, a good relationship was observed between helium concentration and the peak area ratio of helium-to-neon-21, with a correlation coefficient of 0.9999. The limit of detection (LOD; S/N ≥ 3) and limit of quantification (LOQ; S/N ≥ 10) of helium were 1.8 and 6.0 ppm, respectively. The LOD value of 1.8 ppm for the present neon-21-aided helium assay method was much lower than that reported by Malbranque et al. (LOD approximately 27 ppm in blood [15]).

Naturally existing neon is present in air at a concentration of 18.18 ppm [16], including the stable isotopes neon-20, neon-21, and neon-22, with natural isotopic ratios of 0.9048, 0.0027, and 0.0925, respectively [17]. In this study, neon-21 was selected as the appropriate IS among the neon isotopes because of its abundance in air that was compatible to the peak intensities of the target helium that ranged from 10 to 1000 ppm (Fig. 5). In addition, reproducible peak detection of neon-21 [peak area units: 70,834 ± 639; *n *= 7; relative standard deviation (RSD), 0.9%] supported the beneficial use of neon-21 as IS.

Intraday and interday repeatabilities of the present helium assay were investigated in whole blood samples spiked with helium. A commercially available human whole blood sample (no. 12081545) was subdivided in vacuum blood collection tubes, and helium gas was spiked by bubbling at concentrations of 100 and 500 ppm in air. As summarized in Table 1, adequate intraday and interday repeatabilities with RSDs of < 5.1% at both helium concentrations were obtained by the present neon-21-aided GC–SIM-MS method.

**Table 1:** Intraday and interday repeatabilities of the present method in whole blood specimens

Spiked	Intraday			Interday		
	Concentration found (ppm)^a^	*n*	Repeatability (% RSD)^b^	Concentration found (ppm)^c^	*n*	Repeatability (% RSD)
Low concentration (100 ppm)	106 ± 4.5	5	4.2	105 ± 5.4	9	5.1
High concentration (500 ppm)	506 ± 8.8	5	1.7	506 ± 6.8	9	1.3

^a^Data are given as mean ± standard deviation

^b^Relative standard deviation

^c^The measurements were made on three consecutive days with triplicate determinations each

### Application of the method to the quantitative analysis of helium in the blood of deceased humans

**Fig. 6:**
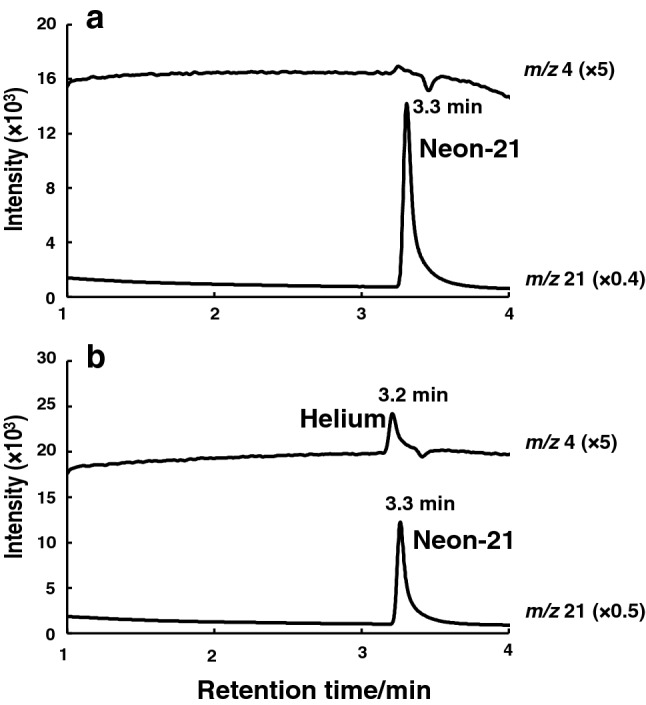
SIM chromatograms of **a** blank blood and **b** the whole blood sample of the deceased in helium inhalation case 1

By using the present GC–SIM-MS method on a double-capillary column, we attempted to determine the amount of helium in heart blood of the humans who died of suffocation as a result of inhalation of helium gas in four cases. A commercially available human whole blood sample was used as the blank specimen (no. 12081545). After 30 min-incubation of the blood in a vacuum blood collection tube at 25 °C, the headspace in the tube was sampled by a gas-tight syringe fitted with a push-pull valve, and injected into the GC–SIM-MS system. As a result of the neon-21-aided SIM analysis of the blood of the deceased, the concentration of helium was successfully determined to be 24 ppm for the heart blood sample in case 1 (Fig. 6b), while almost no peak of helium was observed in the blank blood (Fig. 6a). In this case, the heart blood sample was collected from the deceased postmortem about 4 days after death and was refrigerated at 4 °C until helium analysis. Analysis of helium was carried out about 120 days after sample collection, because it took time to develop the present assay method.

In the other three cases, the concentration of helium was determined to be 90, 138, and 497 ppm. The three blood samples of the deceased were obtained at day 1 or a few days after death from the heart postmortem. Each sample was refrigerated at 4 °C and assayed about 20–100 days after sample collection.

The helium level of ≥ 24 ppm in the blood of the helium-suffocated deceased was in the range of the reported levels of 27 ppm [15] and 400 ppm [11]. According to Henry’s law [19], the ratio of helium-to-neon-21 in the headspace must be matched with that in the blood matrix for any reduced pressure of the vacuum blood collection tube, because both the inert targets were rapidly equilibrated between the headspace and blood matrix. Considering the method and results together, the proposed GC–SIM-MS method with the aid of neon-21 as the IS seems valid for quantitative helium assays in blood. In addition, the use of neon-21 showing a constant blood level by natural breath must be of great benefit in forensic science, because the immersed helium-to-neon-21 ratio in the tube is fixed at the time of blood sampling; then, the ratio in the vacuum tube is constant, without any influence of the storage conditions such as period and temperature.

## Conclusions

In this study, we developed a novel quantitative blood helium assay method by GC–SIM-MS with the aid of neon-21 as IS. In order to determine both helium and neon-21 content, the GC–SIM-MS system was equipped with a double-TC-Molsieve 5A capillary column system. Under the optimized conditions, with hydrogen as the carrier gas at a flow rate of 36 cm/s and a 1.0-kV detector voltage, a highly sensitive and reproducible helium assay could be achieved, with an LOD of 1.8 ppm and RSD not greater than 5.1%. The amount of helium in the headspace of the vacuum blood collection tube containing each whole blood of the four deceased after helium inhalation was successfully determined to be 24–497 ppm based on the present method. It was thus concluded that the GC–SIM-MS method in combination with the naturally existing neon-21 as IS may be extensively applicable to the quantitative assays of stable gases in biological samples, without tedious procedures.
